# 18‐FDG PET in the Evaluation of Therapeutic Response of Necrotizing Otitis Externa

**DOI:** 10.1002/lary.70102

**Published:** 2025-09-02

**Authors:** Lucie Lécolier, Philippine Toulemonde, Damien Huglo, Clio Baillet, Pauline Thill, Gauthier Delaby

**Affiliations:** ^1^ Nuclear Medicine Department Claude Huriez Hospital Lille France; ^2^ Otolaryngology Department Roger Salengro Hospital Lille France

**Keywords:** antibiotherapy, FDG, necrotizing otitis externa, PET scanner

## Abstract

**Objective:**

The duration of antibiotic treatment for necrotizing otitis externa (NEO) and the criteria for discontinuation are currently uncodified. Our study evaluated the performance of [18F]FDG PET to assess the therapeutic response of NEO.

**Methods:**

We retrospectively included patients treated for NEO who underwent a PET scan at baseline and 4 to 9 weeks after antibiotic initiation. PET images were analyzed visually and quantitatively (SUVmax, Total Lesion Glycolysis [TLG], and Total Metabolic Volume [TMV]). An intensity‐weighted extent score (extent) that we created was also calculated. For each parameter, delta values (“∆”: percentage change from baseline for each parameter) were also calculated.

**Results:**

Thirty‐nine patients and 40 ears were enrolled. *Pseudomonas aeruginosa* was isolated from 32 (86%) samples. Twenty‐nine (72.5%) patients were treated with CEFTAZIDIME and CIPROFLOXACIN for a median of 6.9 weeks. On PET scan re‐evaluation, 15 (27.5%) patients were visually in complete response, 17 (42.5%) in partial response, 5 (12.5%) stable, and 3 (7.5%) in progression. The sensitivity and specificity of visual analysis for the diagnosis of recovery were 100% and 43%, respectively. The best performing parameters for predicting recurrence were initial TLG and TMV (sensitivity and specificity of 80% and 100% for respective thresholds of 50.1 g and 8.3 mL). ∆extent showed sensitivity and specificity of 80% and 97.1% using a threshold of −4.3%, and ∆TLG showed sensitivity and specificity of 80% and 94.3% using a threshold of −37.4%.

**Conclusion:**

PET performed well in assessing the therapeutic response of NEO, with excellent sensitivity but low specificity of visual analysis. A complementary quantitative analysis was useful to distinguish between satisfactory and insufficient partial responses.

**Level of Evidence:**

Level 3.

## Graphical abstract

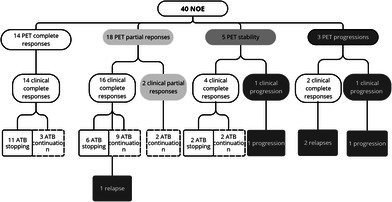

## 1 Introduction

Necrotizing otitis externa (NOE), also known as malignant otitis externa, is a rare and serious infection that initiates in the external auditory canal (EAC). It then spreads through the fissures of Santorini to the soft tissues and bones, progressing to a skull base osteomyelitis [1, 2, 3, 4, 5]. It mainly affects elderly, diabetic, or immunocompromised patients. It is caused in most cases (50% to 90%) by *Pseudomonas aeruginosa* , a saprophytic and opportunistic gram‐negative bacillus, capable of developing multiple antibiotic resistances [1].

The clinical presentation mainly associates severe otalgia, purulent otorrhoea, hypoacusis, and sometimes altered general condition. General infectious signs such as hyperthermia are rare, and a biological inflammatory syndrome is inconsistent [1]. Homolateral cranial nerve deficits are common. The facial nerve is most affected by infiltration of the stylomastoid foramen, causing peripheral facial paralysis, but other cranial nerves may be involved. Otoscopy reveals an inflamed duct, sometimes stenotic, often associated with granulation tissue or polyps, which is highly specific for NOE [4, 6]. In addition to the clinical aspect, refractoriness to conventional treatment is a major argument for questioning the diagnosis of simple otitis externa. Identification of the causal infectious agent must be a priority before antibiotic therapy is initiated, as soon as clinical suspicion arises. Superficial samples of the EAC are obtained primarily by swabbing an otorrhoea or sampling a polyp/granulation tissue sample and may be supplemented by deep sampling under general anesthetic.

Imaging also plays an important role in diagnosis [7]. Computed tomography (CT) is the first‐line modality, demonstrating the bone lysis characteristic of malignant otitis externa, which occurs after demineralization of 30%–50% of the trabecular bone, approximately 2 weeks after the onset of infection [8]. MRI is complementary and allows a better assessment of the soft tissues and the search for intracranial complications.

Treatment is based on a prolonged bi‐antibiotic therapy probabilistically targeting *Pseudomonas aeruginosa* initially (generally an association of a 3rd generation cephalosporin with a quinolone: CEFTAZIDIME and CIPROFLOXACIN), then adjusted according to the antibiogram. The duration of antibiotic therapy is usually at least 6 weeks but is not codified [9, 10]. The assessment of therapeutic response in patients with NOE is a major challenge. The diagnosis of cure is based on a combination of clinical, biological, and imaging evidence. However, there are currently no truly validated criteria to confirm cure. Regression of clinical signs and improvement of otoscopic appearance are the most important criteria (although nerve palsies may not recover). A common limitation of CT and MRI is the prolonged persistence of abnormalities after cure, which limits their use in assessing response to treatment [7, 11, 12].

As early as the 1980s, nuclear medicine studies demonstrated their value in the management of NOE. Technetium‐99m‐labeled diphosphonate bone scintigraphy and gallium‐67 citrate scintigraphy have traditionally been used for early diagnosis of osteitis and therapeutic follow‐up [13, 14, 15, 16, 17, 18]. However, these modalities have limitations in terms of specificity and spatial resolution and are now being abandoned in favor of higher quality investigations [19, 20].

In a few small retrospective studies, labeled white blood cell scintigraphy has shown good diagnostic sensitivity and potential for assessing therapeutic response [21, 22, 23, 24, 25]. In practice, however, it is limited by its modest spatial resolution, time‐consuming radiotracer preparation, two‐day turnaround time, and limited access to most nuclear medicine centers.

More recently, 2‐[18F]fluoro‐2‐deoxy‐D‐glucose ([18F]FDG) PET has emerged as a potentially superior technique, offering better spatial resolution, a shorter protocol, and accessibility to the majority of nuclear medicine centers. It quickly demonstrated its interest and high sensitivity for the diagnosis of active infection [26]. A number of studies have demonstrated its satisfactory diagnostic performance for the diagnosis of NOE [8, 20]. In a retrospective study of 77 patients, Kulkarni et al. [27] found a sensitivity of 97% and a specificity of 93% for the diagnosis of NE0. The value of PET in assessing response to therapy has also been demonstrated in a few small studies, with encouraging results [23, 27, 28]. Kulkarni et al. [27] showed that PET correctly identified progression or improvement in 100% of 23 patients. In a retrospective study of 11 patients, Vion et al. [23] found a sensitivity and specificity of 43% and 100% for the diagnosis of cure for [18F]FDG PET and 86% and 75% for labeled leukocyte scintigraphy. More recently, Hurstel et al. [29] found an accuracy of 100% of [18F]FDG PET to predict patient recovery using the SUV‐lesion‐to‐background with a cut‐off of 4.1.

The aim of this study was to further evaluate the performance of [18F]FDG PET compared to clinical practice in the assessment of therapeutic response to antibiotic therapy in NOE and to identify interpretation parameters to best guide physician decision making in antibiotic therapy management.

## 2 Materials and Methods

### 2.1 Study Population

This was a single‐center retrospective study which included patients with a diagnosis of NOE made between 2016 and 2024, who underwent a [18F]FDG PET scan at initial assessment and then 4 to 9 weeks after the start of antibiotic therapy. This project complies with the reference methodology MR‐004 and has been registered to the record of processing activities in accordance with Article 30 of the EU Regulation (2016/679) (reference number DEC24‐019). Because this was a retrospective study, the requirement to obtain signed informed consent was waived. Clinical follow‐up included a clinical examination close to the re‐evaluation PET scan and a follow‐up of at least 3 months to ensure the absence of early recurrence. At initial assessment, the following parameters were collected: age at the start of treatment, sex, diabetes, or other immunosuppressive etiology, clinical symptoms (altered general condition, otorrhoea, otalgia, headache, hypoacusis, cranial nerve palsies) and biological parameters (CRP, blood leukocyte levels, β‐D‐glucans, galactomannans and glycated hemoglobin). The type of microbiological sampling (superficial/deep) and the results of the bacteriological analyses were recorded, together with the duration and the type of antibiotic treatment.

At re‐evaluation, clinical symptoms were classified into four groups: complete response, partial response, stability, and deterioration. Complete clinical response was defined as complete healing of the EAC on otoscopy and resolution of clinical symptoms other than facial paralysis. After this clinical reassessment, the decision to stop, continue, or change antibiotic therapy was recorded.

### 2.2 PET Scans

[18F]FDG PET scans were performed on a variety of machines, the majority of which were Biograph mCTs (Siemens Medical Solutions) for 66 scans. Patients fasted for 6 h before intravenous injection of 3–4 MBq/kg of [18F]FDG. Low‐dose CT acquisition from the vertex to the mid‐thigh was performed 60 min after tracer administration, without injection of iodine contrast enhancement. PET images were reconstructed using an ordered subsets expectation maximization (OSEM) iterative reconstruction algorithm, with CT images used for attenuation correction.

### 2.3 PET Image Interpretation

Image processing was performed on a single Siemens Syngo.Via console. A total of 80 PET scans were analyzed, blinded to clinical data and double‐read. Images were initially analyzed visually at initial and re‐evaluation. NOE were classified as complete response, partial response, stability, and progression on PET according to the visual change in extent and intensity of metabolic uptake. Complete response was defined as an uptake intensity less than or equal to hepatic activity, partial response as an uptake decrease remaining greater than hepatic activity, progression as an increase in extent and/or uptake intensity, and stability as no visual change. Semi‐quantitative binding parameters were then measured.

Maximum uptake intensity was measured by standardized uptake value (SUVmax).

Total metabolic volume (TMV) was defined as the sum of the volume of all voxels with intensity above a threshold set at 4.0 g/mL in the region of interest. Total lesion glycolysis (TLG) was calculated by multiplying TMV by the mean SUV in the region of interest. The percentage reduction of each metric in relation to initial PET was calculated (Δ).

An analysis by anatomical region was also performed, with a total of 12 spaces: EAC, middle ear, inner ear, temporomandibular joint, mastoid, petrous apex, carotid space, parapharyngeal space, retropharyngeal space, deep cervical space, clivus, and intracranial space. A region was considered positive if there was any abnormal fixation of any intensity. Hypermetabolism related to residual inflammation from a recent deep biopsy was excluded. NOE were classified as superficial if hypermetabolism was limited to the EAC, middle ear, and/or mastoid, and as deep if hypermetabolism extended to at least one of the other spaces.

A 5‐point fixation intensity score was generated for each area:

– 0: no uptake or symmetry with the contralateral region.
– 1: SUVmax less than the vascular SUVmax defined in the descending aorta.
– 2: SUVmax between vascular SUVmax and hepatic SUVmax,
– 3: SUVmax greater than or equal to hepatic SUVmax.
– 4: SUVmax greater than twice the hepatic SUVmax.

A weighted intensity and extent score was generated, named “extent score” calculated as a percentage equal to the sum of the fixation intensity scores for each space divided by 48 (12 spaces; score from 0 to 4). The relative changes of all parameters (SUV, TMV, TLG, extent score) between initial and re‐evaluation PET were computed as the decrease in percentage [e.g., Δparameter = (reevaluation − initial)/initial × 100)].

### 2.4 Statistical Analysis

Statistical analyses were performed using SPSS software (IBM Corp. 2011, IBM SPSS Statistics for Windows, version 20.0. Armonk, NY: IBM Corp.). Qualitative data were presented as numbers and percentages. Normality of quantitative data was tested using the Shapiro–Wilk test. Quantitative data were expressed as mean and standard deviation in the case of a normal distribution, and as median and range in the case of a non‐normal distribution. The distribution of the two groups was compared using Student's *t*‐test for normal distribution and non‐parametric Mann–Whitney test for non‐normal distribution. Receiver operating characteristic (ROC) curves were analyzed to assess the diagnostic performance of the semi‐quantitative parameters. The maximum Youden index was used to select the optimal threshold. A *p*‐value < 0.05 was considered significant.

## 3 Results

### 3.1 Population

Thirty‐nine patients were included. One patient (2.5%) presented with contralateral recurrence 4 years later and was considered as two separate events. Another (2.5%) had bilateral involvement. Forty ears were examined. The baseline clinical and biological characteristics of the patients were summarized in Table 1. All but one patient had otalgia and/or headache at diagnosis. This patient had limited involvement of the CAE and the deep cervical space. Nine patients (23.1%) had facial nerve paralysis. Eight of them had involvement of the carotid space (4 of them with a mastoid involvement associated) and the ninth had only mastoid involvement. It is to be noted that 14 patients had carotid space and/or mastoid involvement without facial nerve paralysis.

**TABLE 1:** Patient characteristics.

Patients	
Sex (male)	32 (81.6%)
Age	76 ± 9 years (52–91)
Diabetes	29 (74.4%)
Immunosuppression	5 (12.8%)
Clinical	
Otalgia and/or headache	38 (97.4%)
Otorrhea	34 (87.2%)
Hypoacusis	26 (66.7%)
Altered general condition	11 (28.2%)
Nerve paralysis	10 (31.3%)
Facial nerve paralysis	9 (23.1%)
Vagus nerve paralysis	1 (2.5%)
Paralysis of oculomotor nerves III, IV and VI	1 (2.5%)
Biology	
CRP increased (> 6 mg/L)	26 (76.4%)^a^
Hyperleukocytosis (10,000/mm^3^)	9 (26.5%)^a^
Galactomannans positive (≥ 0.5)	0 (0%)^a^
β‐D‐1,3‐glucans positive (> 80 pg/mL)	2 (9.5%)^a^
Areas affected on initial PET scan	
External auditory canal	38 (95%)
Middle ear	23 (57.5%)
Mastoid	17 (42.5%)
Carotid space	17 (42.5%)
Deep cervical space	17 (42.5%)
Temporomandibular joint	10 (25%)
Petrous apex	9 (22.5%)
Retropharyngeal space	8 (20%)
Parapharyngeal space	7 (17.5%)
Clivus	5 (12.5%)
Intracranial	5 (12.5%)
Contralateral involvement through crossing of midline	2 (5%)
Inner ear	0 (0%)

*Note*: Categorical variables are expressed as headcount and proportion. Quantitative variables are expressed as mean, standard deviation and range.

^a^ As a percentage of available data.

### 3.2 Microbiology and Antibiotic Therapy

Bacteriological samples were obtained from 37 ears (92.5%). The sample was superficial only in 19 cases (51.4%) and superficial and deep in 18 cases (48.6%). In three patients, no samples were taken. Thirty‐two samples (86%) were positive for *Pseudomonas aeruginosa* , isolated in 26 cases (81.3%) and polymicrobial in the others. Of these, 28 (87.5%) were susceptible to CIPROFLOXACIN, CEFTAZIDIME, and CEFEPIME. The median duration of antibiotic treatment was 6.9 weeks [5.4–14.6 weeks]. Twenty‐nine patients (72.5%) were treated with CEFTAZIDIME and CIPROFLOXACIN. The fungi found in the samples were not considered pathogenic and were not initially targeted by antibiotic therapy. The results of microbiological sampling and the different therapeutic combinations were summarized in Table 2.

**TABLE 2:** Microbiology and initial antibiotherapy.

Microbiology		*n* (%)	Initial antibiotherapy
*Pseudomonas aeruginosa* isolate	Susceptible	22 (59.5%)	19 CEFTAZIDIME and CIPROFLOXACIN 2 CEFEPIME and CIPROFLOXACIN 1 CEFEPIME and DAPTOMYCIN
	Resistant to CIPROFLOXACIN	3 (8.1%)	2 CEFTAZIDIME 1 CEFTAZIDIME and LINEZOLIDE
	Resistant to CIPROFLOXACIN and CEFEPIME	1 (2.7%)	CEFTAZIDIME
*Pseudomonas aeruginosa* susceptible + *MSSM*		3 (8.1%)	2 CEFTAZIDIME and CIPROFLOXACIN 1 CEFEPIME and LEVOFLOXACIN 6 weeks then CLINDAMYCINE and LEVOFLOXACIN 6 weeks
*Pseudomonas aeruginosa* susceptible + *Staphylococcus lugdunensis*		1 (2.7%)	CEFEPIME, RIFAMPICIN and DOXYCYCLINE
*Pseudomonas aeruginosa* susceptible + *Candida parapsilosis*		1 (2.7%)	CEFTAZIDIME and CIPROFLOXACIN
*Pseudomonas aeruginosa* susceptible + *MSSM* + *Streptococcus pneumoniae*		1 (2.7%)	PIPERACILLIN‐TAZOBACTAM, LEVOFLOXACIN and FLUCONAZOLE
*MSSM* + *Aspergillus fumigatus*		1 (2.7%)	CEFTAZIDIME and CIPROFLOXACIN
*Candida parapsilosis*		1 (2.7%)	CEFTAZIDIME and CIPROFLOXACIN
*Enterococcus faecalis*		1 (2.7%)	AMOXICILLINE and CEFTRIAXONE
Non‐contributory		2 (5.4%)	CEFTAZIDIME and CIPROFLOXACIN

Abbreviation: MSSM, Methicillin‐susceptible *Staphylococcus aureus* .

### 3.3 Initial PET Scan

Twenty‐five patients (62.5%) had their initial PET scan before starting antibiotherapy, with a median of 4 [0 to 19] days. Fifteen patients (37.5%) had their first PET scan after the start of antibiotic therapy, with a median of 5 [2 to 10] days. Seven patients had deep sampling prior to the first PET scan, with a median delay of 7 [2 to 23] days. All PET scans were positive at initial assessment. The SUVmax was always greater than liver activity (score ≥ 3) and was greater than twice liver activity (score = 4) in 30 patients (75%), with a median SUVmax of 9.4 [3.4 to 21.9] g/mL. The median TLG was 28.3 [0 to 273] g. The median TMV was 4.8 [0 to 45.2] mL. Ten patients (25%) had superficial involvement and 30 patients (75%) had deep involvement. Five patients (12.5%) had intracranial extension. Two patients had contralateral midline crossing. Hypermetabolic involvement averaged 4 out of 12 regions ±2.5 [1 to 11 regions]. The extent score had a median value of 20.8 [6.3 to 66.7]%.

### 3.4 PET and Clinical Follow‐Up

**FIGURE 1:**
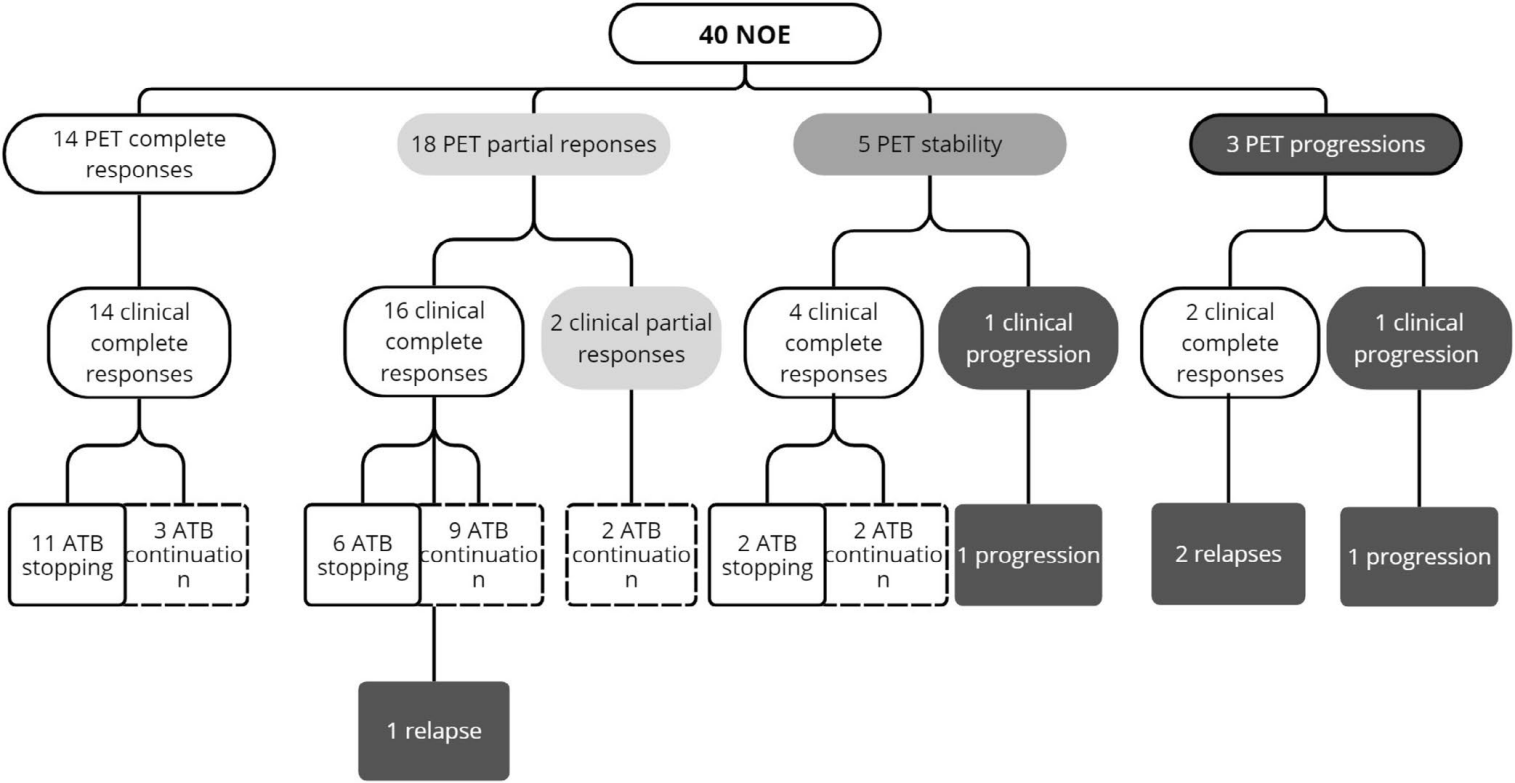
Classification of patients included for reassessment according to PET response, clinical response and clinical course. ATB, antibiotherapy.

**FIGURE 2:**
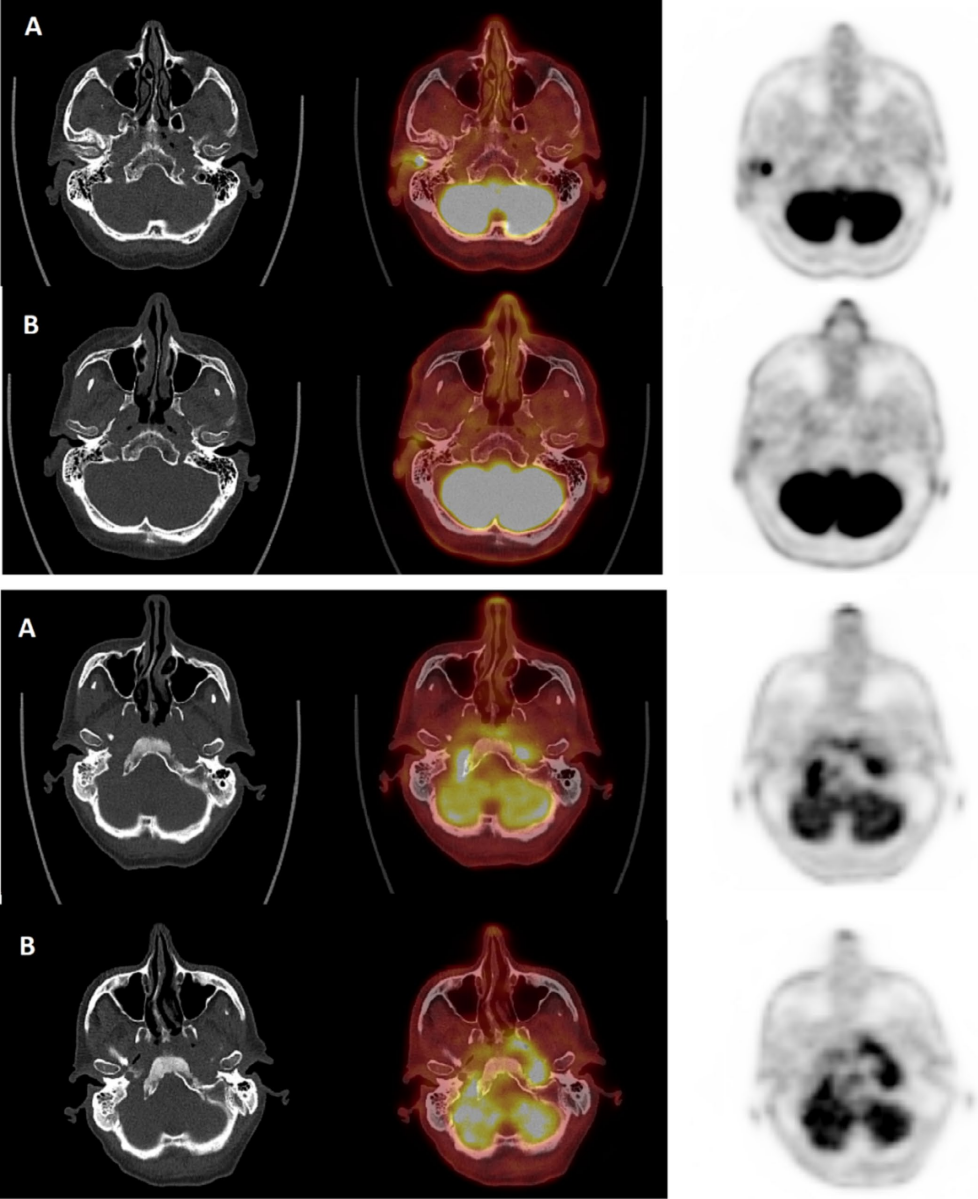
(a) Example of patient who have achieved a cure. A: initial PET scan; B: re‐evaluation PET scan; from left to right, in axial section: CT—PET‐CT fusion—PET alone. NOE with susceptible *Pseudomonas aeruginosa* and *MSSM*, treated with CEFTAZIDIME and CIPROFLOXACIN for 7 weeks. A: Intense uptake centered on the right temporomandibular joint, extending to the external auditory canal and middle ear, with erosion of the mandibular condyle and floor of the tympanal bone (SUVmax 21.9). B: Partial regression in intensity and extent of uptake (SUVmax 5.6; ∆TLG: −99%; ∆VMT: −85%; ∆extent: −54%). Good clinical evolution thereafter. (b) Example of patient with early progression or recurrence. A: initial PET scan; B: re‐evaluation PET scan; from left to right, in axial section: CT—PET‐CT fusion—PET alone. NOE with *MSSM* and *Aspergillus fumigatus* , treated with CEFTAZIDIME and CIPROFLOXACINE for 6 weeks. A: Intense uptake of bilateral parapharyngeal, carotid, and retropharyngeal spaces, with extension along the right styloid process, right petrous apex, and right jugular foramen. Erosion of the anterior cortex of the clivus (SUVmax 7.3). B: Progression in extent and stability in intensity of hypermetabolic involvement (SUVmax 7.1; ∆TLG 60%; ∆VMT 29%; ∆extent 0%). Clinical worsening thereafter.

At re‐evaluation after 6 weeks of antibiotic therapy, 34 patients (85%) had a complete clinical response, 3 (7.5%) had partial improvement, and 3 (7.5%) had worsening symptoms. Re‐evaluation PET scans were performed a median of 6.1 [4.3 to 8] weeks after the start of antibiotic therapy. When the PET scans were re‐evaluated, 14 patients (35%) had a complete response, 18 (45%) had a partial response, 5 (12.5%) had a stable response, and 3 (7.5%) had a progressive response. The median SUVmax was 5.6 [2.1 to 14.7] g/mL. The median TLG was 14.7 [0 to 174] g. The median TMV was 1.1 [0 to 28] mL. The distribution of various PET and clinical responses and patient outcomes is summarized in Figure 1, and examples of images at initial assessment and re‐assessment are shown in Figure 2.

The 14 patients with a complete response on PET had a complete clinical response, and none of them relapsed. It should be noted that the two patients with *Candida* treated with CEFTAZIDIME and CIPROFLOXACIN had a complete response on PET, and neither relapsed without antifungal treatment.

Of the 18 patients with a partial response on PET, 16 had a complete clinical response and only one relapsed. This patient was infected with CIPROFLOXACIN‐resistant *Pseudomonas aeruginosa* and treated with CEFTAZIDIME and LINEZOLIDE for 44 days. Two had a partial clinical response (persistent otalgia and inflammatory CAE) and did not relapse.

Of the five patients who were stable on PET, one was clinically progressive and continued to deteriorate (worsen otorrhea and altered general condition). He was infected with sensitive *Pseudomonas aeruginosa* and treated with CEFTAZIDIME and CIPROFLOXACIN. CEFEPIME and METRONIDAZOLE were then initiated, with favorable evolution. Four patients had a complete clinical response and none of them relapsed.

Of the three patients who progressed on PET, two had a clinical complete response but relapsed early, and one was clinically progressive and continued to deteriorate (worsen hypoacousis, vestibular disorders, contralateral otitis). One of these patients was infected with CIPROFLOXACIN‐resistant *Pseudomonas aeruginosa* treated with CEFTAZIDIME alone. One other did not have any microbiological sampling and was treated probabilistically with CEFTAZIDIME and CIPROFLOXACIN for 42 days, and then relapsed 1 month later (swab found *Pseudomonas aeruginosa* resistant to CEFTAZIDIME and CIPROFLOXACIN). The last one was infected with *Aspergillus fumigatus* and *SAMS* and was treated with CEFTAZIDIME and CIPROFLOXACIN.

Of all patients, 15 were still on their initial antibiotic therapy after reassessment, including 3 who had a complete response on PET, 11 who had a partial response, and 1 who was stable on PET.

Table 3 summarizes the microbiology and the antibiotic treatment given to the five patients who relapsed, at initial assessment and at relapse.

**TABLE 3:** Microbiology and treatment of patients not cured.

Patient no.	Initial			Relapse			
	Type of sampling	Microbiology	Treatment	Type of sampling	Microbiology	Treatment	Evolution
24	Superficial	*Pseudomonas aeruginosa* resistant to Ciprofloxacin	Ceftazidime 5.6 weeks	Superficial and deep	Non contributive	Meropenem	Pancytopenia and progression
11	None	None	Ceftazidime and Ciprofloxacin 6 weeks	Deep	*Pseudomonas aeruginosa* resistant to Ceftazidime and Ciprofloxacin	Colimycin and Meropenem	Favorable
14	Superficial	*Aspergillus fumigatus* and *SAMS*	Ceftazidime and Ciprofloxacin 6.4 weeks	Superficial and deep	*Aspergillus fumigatus*	Meropenem Zyvoxid and Voriconazole	Favorable
18	Superficial and deep	*Pseudomonas aeruginosa* susceptible	Ceftazidime and Ciprofloxacin 6.7 weeks	Superficial	Non contributive	Cefepime and Metronidazole	Favorable
21	Superficial	*Pseudomonas aeruginosa* resistant to Ciprofloxacin	Ceftazidime and Linezolid 6.3 weeks	None	None	Ceftazidime	Favorable

Abbreviation: MSSM, Methicillin‐susceptible *Staphylococcus aureus* .

In the group of 21 patients, combining patients with a partial response and patients who were stable on PET and had not relapsed, there was no significant difference in ΔTMV (*p* = 0.432), ΔTLG (*p* = 0.347), ΔSUVmax (*p* = 0.426), or ΔExtent (*p* = 0.765) between patients in whom antibiotic therapy was stopped (*n* = 9) or continued (*n* = 12). Of these patients, 14 had a follow‐up PET scan approximately 3–4 months after the start of antibiotic therapy (median 3.0; 2.5 to 4.7 months) and only five of these were considered negative.

### 3.5 Quantification at Initial PET Scan

The median initial TLG, TMV, and extent were significantly higher in patients who had failed therapy compared to those who had not relapsed (TLG 64.5 [32.4 to 273.4] g versus 22.8 [0 to 130] g; *p* = 0.003—TMV 13.5 [8.25 to 45.2] mL versus 3.9 [0 to 17.1] mL; *p* = 0.0001—Extent 54.2 [29.2 to 66.7]% versus 18.8 [6.3 to 50.0]%; *p* = 0.001). However, there was no significant difference in initial SUVmax between patients who relapsed and those who did not (7.6 [7.3 to 15.9] g/mL versus 9.5 [3.4 to 21.9] g/mL; *p* = 0.968). All patients who relapsed had initial deep involvement, compared with 71.4% of patients who were cured (25/35 cured patients).

### 3.6 Diagnostic Performance of Follow‐Up PET Scans

Visual analysis alone, considering complete response as maximal intensity less than liver activity, predicted cure with 100% sensitivity, 42.9% specificity, and 50.0% accuracy. The SUVmax, TLG, TMV, Extent, ∆SUVmax, ∆TLG, ∆TMV, and ∆Extent were all significantly lower in patients who did not relapse than in those who did. The summary of quantitative data at baseline and at recurrence are shown in Table 4. The diagnostic performance values of the different quantitative data are summarized in Table 5. The best performing parameters for predicting recurrence were TLG and TMV with sensitivity and specificity of 80% and 100% using respective thresholds of 50.1 g and 8.3 mL. ∆Extent showed a sensitivity and specificity of 80% and 97.1% using a threshold of −4.3% (i.e., a decrease of approximately two points on the sum of intensity scores). ∆TLG and ∆SUVmax showed a sensitivity and specificity of 80% and 94.3% using thresholds of −37.4% and −4.4%, respectively, then ∆TMV with a sensitivity and specificity of 80% and 82.9% using a threshold of −45.5%.

**TABLE 4:** Quantification on initial and re‐evaluation PET scans.

	Initial PET scan	Re‐evaluation PET scan			Progression
		Complete response	Partial response	Stability	
*n* (%)	40 (100%)	14 (35%)	18 (45%)	5 (12.5%)	3 (7.5%)
SUVmax	9.4 [3.4 to 21.9] g/mL	3.1 [2.1 to 4.6] g/mL	6.1 [3.3 to 8.3] g/mL	9.5 [6.7 to 13.3] g/mL	11 [7.1 to 14.7] g/mL
TLG	28.3 [0 to 273] g	0 [0 to 0.9] g	0 [0 to 14.6] g	15.2 [13.4 to 173.8] g	53.4 [51.7 to 129] g
TMV	4.8 [0 to 45.2] mL	0 [0 to 0.04] mL	0 [0 to 4.4] mL	3.2 [2.6 to 28.2] mL	10.6 [8.7 to 22.8] mL
Extent	20.8 [6.3 to 66.7]%	4.2 [0 to 6.3]%	14.6 [0 to 41.7]%	22.9 [10.4 to 64.6]%	41.7 [35.4 to 79]%
SUVmax		−56.8 [−78 to −31.9]%	−33.4 [−74.6 to −6.4]%	−0.3 [−31.4 to 1.5]%	47.5 [−2 to 92]%
TLG		−100 [−100 to 0]%	−84.7 [−100 to −3.8]%	−39.4 [−53.8 to 67.8]%	59.7 [21 to 100]%
TMV		−100 [−100 to 0]%	−81.2 [−100 to −3.1]%	−35.1 [−54.1 to 69.1]%	28.7 [0.5 to 68.5]%
Extent		−70.8 [−100 to −33.3]%	−48.5 [−100 to −15.4]%	−0.55% [−11.1 to 0]%	21.4 [0 to 46]%

*Note*: Quantitative variables are expressed as median and range. Qualitative variables are expressed as headcount and percentage of the population.

Abbreviations: SUV, standardized uptake value; TLG, total lesion glycolysis; TMV, total metabolic volume.

**TABLE 5:** Quantitative parameters on PET according to clinical course.

	Recovery	Relapse	*p*
Initial parameters			
SUVmax	9.5 [3.4 to 21.9] g/mL	7.6 [7.3 to 15.9] g/mL	0.968
TLG	22.8 [0 to 130] g	64.5 [32.4 to 273.4]g	0.003
TMV	3.9 [0 to 17.1] mL	13.5 [8.3 to 45.2] mL	< 0.001
Extent	18.8 [6.3 to 50.0]%	54.2 [29.2 to 66.7]%	0.001

	Recovery	Relapse	*p*	Threshold	AUC	Se	Sp
Parameters at re‐evaluation							
SUVmax	5.4 [2.1 to 13.3] g/mL	11.0 [5.5 to 14.7] g/mL	0.008	7.0	0.857	80%	82.9%
TLG	1.7 [0 to 48.5] g	53.4 [6.8 to 173.8] g	> 0.001	50.1	0.937	80%	100%
TMV	0.5 [0 to 8.0] mL	10.6 [1.6 to 28.2] mL	> 0.001	8.3	0.943	80%	100%
∆SUVmax	−40.8 [−78.0 to 0.4]%	−1.5 [−65.7 to 92.4]%	0.021	−4.4%	0.817	80%	94.3%
∆TLG	−90.8 [−100 to −67.8]%	−21.1 [−94.6 to 100]%	0.014	−37.4%	0.869	80%	94.3%
∆TMV	−85.2 [−100 to −69.1]%	−0.5 [−92.7 to 68.5]%	0.006	−45.5%	0.834	80%	82.9%
∆Extent	−54.5 [−100 to 0]%	0 [−57.7 to 46.2]%	0.003	−4.3%	0.889	80%	97.1%

*Note: p* significant if less than 0.05.

Abbreviations: Se, sensitivity; Sp, specificity; SUV, standardized uptake value; TLG, total lesion glycolysis; TMV, total metabolic volume.

## 4 Discussion

The duration of antibiotic treatment for necrotising otitis externa is not yet codified and the decision to stop or continue antibiotic therapy is a complex one based on several clinical, biological, and imaging factors. At present, there is no standardized criterion to determine cure with certainty. [18F]FDG‐PET is used in routine practice in many centers, but the literature evaluating its performance in assessing therapeutic response is poor and limited to a few retrospective studies with small numbers [23, 27, 28]. Our aim was to evaluate its performance in a larger population and to identify interpretation criteria to facilitate clinical decision making.

The patients in our study presented the typical ONE profile, in line with the literature and in particular the systematic review by Takata et al. [10], i.e., predominantly male (82% vs. 68%), diabetic (74.4% vs. 84%), and elderly (76 ± 9 vs. 69.2 years). The clinical presentation was also typical, with the main symptom being otalgia (and/or headache), which was almost constant (97.4%), followed by otorrhoea (87.2%), hypoacusis (66.7%), altered general condition (28.2%) and peripheral facial paralysis (23.1%). As widely reported, *Pseudmonas aeruginosa* was the most isolated germ (86% vs. 62% in the review by Takata et al.) with ciprofloxacin resistance in 10.8% (vs. 8%) of samples.

Firstly, our results highlight that the treatments offered for NOE are now effective, with only 5 out of 40 patients failing treatment (12.5%). All patients had a positive PET scan at baseline, which is consistent with studies by Vion et al. [23] and Kulkarni et al. [27]

One of the most important results of our study was that the 15 patients (100%) with a complete visual response on PET showed a favorable progression, reflecting a specificity of 100% for the diagnosis of cure. Conversely, visual progression on PET was systematically associated with treatment failure (three patients), even in the presence of a good clinical response. This suggests that PET was reliable for identifying cured patients in whom antibiotic therapy can be safely discontinued, and patients with treatment failure in whom a change in antibiotic therapy or new bacteriological samples should be considered. On the other hand, the sensitivity of PET complete visual response for identifying a cure was only 43%. Indeed, more than half of the cured patients had an incomplete (partial or stable) PET response, but only one in 17 partial responses relapsed, suggesting that these are also predominantly associated with a favorable outcome.

These results were in agreement with the retrospective study of 11 patients by Vion et al. [23]. [18F]FDG‐PET/CT was truly positive in 4/4 uncured patients (i.e., sensitivity 100%) and truly negative in 3/7 cured patients (i.e., specificity 43%). Though, lack of complete response was defined in this study as “focal increase of radiotracer compared to the contralateral side,” sometimes with low fixations still considered positive at re‐evaluation (four patients with a SUVmax of less than three considered positive). In our study, a complete response was defined as a residual fixation lower than the hepatic uptake. This hepatic threshold has been widely accepted in the literature as a good physiological reference, particularly in the context of haemopathies (lymphoma) [30] and inflammatory diseases (vasculitis) [31]. It seems to be an easy parameter to use, which allows to avoid an over‐positivisation of low residual hypermetabolisms, which are likely to be of inflammatory origin. It is important to reiterate that this visual analysis remains subjective (particularly the classification of patients as stable, partial response or progression) was not based on strict quantification criteria and remained at the discretion of the observer.

Though PET is not often used in patient follow‐up after antibiotherapy discontinuation, observation of the few surveillance PET scans of our cured patients showed that PET scans regularly remained weakly positive for several months. This residual uptake may be related to a significant inflammatory component in the metabolism of NEO and to scarring phenomena that may be active for a prolonged period. This suggests that the persistence of moderate uptake should not systematically justify prolongation of antibiotic treatment. Reasonably, there seemed to be no justification for PET monitoring until complete negativity, as long as the clinical course was favorable.

In situations where patients are metabolically stable or in partial response, quantification may be of interest to compensate for the lack of sensitivity of visual PET analysis. Indeed, all parameters were significantly higher in patients who had failed therapy than in those with favorable outcomes. Sensitivity was 80% for all parameters. This equivalent sensitivity is explained by the fact that one patient had a significant response for all parameters, improved clinically at reassessment, and yet relapsed early (one false negative out of five patients who relapsed). TLG and TMV had the best specificity of 100%, with respective thresholds of 50.1 g and 8.3 mL. ΔTLG and ΔTMV are also effective and appear to be reliable in doubtful situations, with respective specificity of 94.3% (threshold of −37.4%) and 82.9% (threshold of −45.5%). ΔExtent also showed an excellent specificity of 97.1% for a threshold of −4%, corresponding to a 2‐point decrease in the sum of intensity score with the extent score we defined. The global performance of our quantitative parameters matches those of Hurstel et al. using a different method; their study found an accuracy of 100% based on a SUVmax‐lesion‐to‐background‐threshold of 4.1 on re‐evaluation PET for predicting recovery from NOE. It is to be noted that our extent score is new, homemade, unvalidated, and deserves to be studied in a larger cohort. It was inspired by the Summed Stress Score (SSS) used in cardiology for myocardial scintigraphy, and we felt that the analogy was relevant in terms of weighting different anatomical spaces according to their intensity. We are aware of certain biases in the design of this score. Firstly, the anatomical regions were all given the same weight despite being very different in size (e.g., middle ear versus mastoid). Very high intensities retained an identical score on re‐evaluation if the intensity decreased but remained greater than twice the hepatic metabolism. Inter‐observer reproducibility was uncertain, and obtaining this score was often time‐consuming and difficult to implement in everyday practice.

In current practice, it may be useful to speak of a “satisfactory” response when these quantitative parameters are below their optimal thresholds and of an inadequate response above these thresholds.

These results pave the way for optimizing treatment, in particular by reducing the duration of antibiotics when PET shows a complete or satisfactory response based on quantitative indices, if clinical evolution is favorable. Limiting the prolongation of antibiotic therapy in these patients would limit the emergence of bacterial resistance, reduce the incidence of antibiotic‐related side effects (such as encephalopathy from overdosage) and reduce the costs associated with prolonged treatment. Another promising aspect of our study was that initial disease extension, as measured by TLG, TMV, and our extent score, was significantly greater in patients who subsequently relapsed, whereas this was not the case for initial uptake intensity. One of the particularities in the one partial response patient who relapsed was the extent of his initial involvement (TLG 126.4 g, TMV 21.1 mL, extent 54.2%). It would be interesting to evaluate the prognostic character of these indices.

Our study had several limitations, the first being its retrospective nature. The population and methodology were representative of current practice and literature, with various bacteriology and antibiotic regimens reflecting the diversity of therapeutic approaches observed in practice. The overall sample size was relatively small, with only a small number of treatment failures; the overall performance of our quantification indices should therefore be treated with caution, especially sensitivity. However, the number of patients was satisfactory given the rarity of the disease and compared to previous studies [23, 27, 28]. The choice of our gold standard could also be discussed given the lack of a validated imaging gold standard for therapeutic response in NOE; we chose to use clinical improvement and sustained remission after antibiotic withdrawal as our reference. This pragmatic approach, commonly used in infectious disease studies, reflects real‐world therapeutic success and remained for us the most robust endpoint available.

Another major limitation of our study was that more than half of the patients with an incomplete PET response continued antibiotic therapy at re‐evaluation (11 of 16 patients with a partial response), making it impossible to assess retrospectively whether this continuation was essential for cure. PET may have played a role in this decision, but it was difficult to assess a posteriori the reasons for continuing or stopping antibiotic therapy. What's more, we found no significant difference between the quantitative PET parameters of patients with a partial or stable response who continued or stopped antibiotic therapy that might have motivated this decision. This highlights the uncertainty and heterogeneity of current practice regarding the optimal management of antibiotic therapy at re‐evaluation in the absence of clear decision‐making criteria. The high proportion of patients with prolonged antibiotic therapy after PET re‐evaluation, a fortiori in patients with partial response, made it impossible to assess retrospectively whether this continuation of antibiotic therapy was essential for cure.

Second, as with all PET studies of metabolic volume, the choice of segmentation threshold was controversial. There was no specific recommendation for this choice in the area of infections. We chose a fixed threshold of 4.0 g/mL SUV rather than, for example, one based on SUVmax (which is widely used in onco‐hematology) because it was easier to evaluate a large volume when the damage was extensive, of complex shape, or close to cerebral metabolism. In addition, as fixation intensities were often moderate (median SUVmax 9.4 [3.4 to 21.9] g/mL), using a relative threshold based on SUVmax risked over‐inclusion of weak, non‐pathological fixations (such as residual inflammation). Finally, our study included different PET devices and therefore different reconstructions of the images, which could introduce a bias in the quantification. However, this bias appeared to be minimal given the fact that most of the examinations were performed on Biograph mCT devices (82.5%).

## 5 Conclusion

This study confirms the value of using [18F]FDG PET to assess therapeutic response in necrotising otitis externa. A combination of visual and quantitative analysis appeared to be effective in identifying patients who could be confidently considered cured. PET was reliable in cases of complete response or visual progression. Quantitative indices were used to refine decision making in patients with incomplete response, with thresholds appearing to be effective in stratifying them into satisfactory or unsatisfactory responses. A prospective study would be interesting to validate the promising results of these quantitative indices.

## Conflicts of Interest

The authors declare no conflicts of interest.

## Data Availability

The data that support the findings of this study are available on request from the corresponding author.
